# Risk stratification with explainable machine learning for 30-day procedure-related mortality and 30-day unplanned readmission in patients with peripheral arterial disease

**DOI:** 10.1371/journal.pone.0277507

**Published:** 2022-11-21

**Authors:** Meredith Cox, J. C. Panagides, Azadeh Tabari, Sanjeeva Kalva, Jayashree Kalpathy-Cramer, Dania Daye

**Affiliations:** Department of Radiology, Massachusetts General Hospital, Harvard Medical School, Boston, MA, United States of America; USC Keck School of Medicine: University of Southern California Keck School of Medicine, UNITED STATES

## Abstract

Predicting 30-day procedure-related mortality risk and 30-day unplanned readmission in patients undergoing lower extremity endovascular interventions for peripheral artery disease (PAD) may assist in improving patient outcomes. Risk prediction of 30-day mortality can help clinicians identify treatment plans to reduce the risk of death, and prediction of 30-day unplanned readmission may improve outcomes by identifying patients who may benefit from readmission prevention strategies. The goal of this study is to develop machine learning models to stratify risk of 30-day procedure-related mortality and 30-day unplanned readmission in patients undergoing lower extremity infra-inguinal endovascular interventions. We used a cohort of 14,444 cases from the American College of Surgeons National Surgical Quality Improvement Program database. For each outcome, we developed and evaluated multiple machine learning models, including Support Vector Machines, Multilayer Perceptrons, and Gradient Boosting Machines, and selected a random forest as the best-performing model for both outcomes. Our 30-day procedure-related mortality model achieved an AUC of 0.75 (95% CI: 0.71–0.79) and our 30-day unplanned readmission model achieved an AUC of 0.68 (95% CI: 0.67–0.71). Stratification of the test set by race (white and non-white), sex (male and female), and age (≥65 years and <65 years) and subsequent evaluation of demographic parity by AUC shows that both models perform equally well across race, sex, and age groups. We interpret the model globally and locally using Gini impurity and SHapley Additive exPlanations (SHAP). Using the top five predictors for death and mortality, we demonstrate differences in survival for subgroups stratified by these predictors, which underscores the utility of our model.

## Introduction

Peripheral arterial disease (PAD) of the lower extremities affects over 200 million people worldwide [1] and is associated with significant morbidity and mortality [2,3]. PAD may progress to severe limb ischemia, requiring endovascular interventions or surgical procedures in order to achieve limb salvage. Unfortunately, despite their “minimally invasive” nature, lower extremity endovascular procedures for critical limb ischemia have a significant peri-procedural mortality rate of 0.5%-3% [4], and more than 1 in 6 patients who undergo endovascular revascularization have unplanned readmission within 30 days [5]. Identification of PAD patients at substantially increased risk for procedure-related mortality may be helpful in setting realistic expectations for procedural outcomes, and/or making alterations in the therapeutic plan to decrease the risk of death. Identifying patients at high risk for 30-day unplanned readmission may allow clinicians to focus on patients who would benefit from strategies to avoid readmission such as telephone-based care management [6], home visits [7], partnering with community physicians [8], and more complex, multidisciplinary interventions [9]. Furthermore, explanations of risk at the individual patient level will allow health systems to differentiate between potentially preventable readmissions and readmissions that are likely to occur due to the natural course of vascular disease.

In addition to generating risk scores to guide patient-level medical decision-making, global explanations of death and readmission projections over all patients may also be useful in informing prevention strategies on a large scale. If novel intervenable factors that contribute to increased risk of mortality and readmission can be identified, health systems may be able to selectively target these indicators to decrease mortality and readmission risk. These factors may also serve as targets for future research in the implementation of strategies to reduce procedure-related mortality and unplanned readmission.

Mortality and readmission after revascularization procedures have been studied in many previous cohort studies [5,10–14] using traditional statistical methods such as logistic regression and Cox proportional hazards. Machine learning has the potential to improve upon these methods by finding generalizable predictive patterns in the data without being constrained by limitations of statistical methods such as a priori assumptions and difficulty addressing interactions [15,16]. Machine learning-based software offered by companies such as Viz.ai, Aidoc, Siemens Healthineers, and many others have demonstrated the ability to improve patient care through the use of machine learning [17–19]—therefore, we aim to use machine learning to develop models for the tasks of predicting 30-day procedure-related mortality and 30-day unplanned readmission.

The goal of this study is to develop interpretable machine learning models to stratify 30-day procedure-related death and 30-day unplanned readmission in PAD patients undergoing lower extremity endovascular revascularization procedures. We used the American College of Surgeons National Surgical Quality Improvement Program (ACS-NSQIP)—a large, multihospital database—to develop and evaluate machine learning models for mortality and readmission. We interpreted each model to identify key risk factors. We additionally performed testing to determine the robustness of each model across different demographic groups (race, sex, and age) to ensure that this model is applicable in multiple care settings with divergent patient populations.

## Methods

Four machine learning models were evaluated for their ability to predict 30-day mortality and readmission outcomes: Support Vector Machines (SVM), Random Forests, Extreme Gradient Boosting (XGBoost), and Multilayer Perceptrons (MLP).

Data to develop the model was obtained from the American College of Surgeons National Surgical Quality Improvement Program (ACS-NSQIP) database, a database containing demographics, laboratory results, clinical variables including lab values and disease progression variables, and 30-day postoperative outcomes. This data was collected from electronic health records from approximately 700 hospitals across the United States. The procedures included in this study were performed from the years 2011 to 2018. The ACS-NSQIP dataset excludes minor cases, cases in which the patient was under 18 years old, cases in which the patient has been assigned an ASA score of 6 (brain-death organ donors), cases involving Hyperthermic Intraperitoneal Chemotherapy (HIPEC), trauma cases, transplant cases, cases that exceeded the limit of three of the following procedures for a single patient (Inguinal Herniorrhapy, breast lumpectomy, laparascopic cholecystectomies, TURPS and/or TURBTs), cases beyond the required number specified in the NSQIP site’s contract, returns to the operating room related to an occurrence or complication of a prior procedure, and cases in which the patient already has a NSQIP-assessed procedure entered within the previous thirty days. We subset the data using CPT codes to include only patients who underwent lower extremity endovascular infrainguinal interventions for PAD. The CPT codes for inclusion are listed in S1 Table.

The ACS-NSQIP database also offers a targeted vascular module with pre- and post-operative variables specific to vascular disease and the type of procedure, which was merged with the previously selected cases to provide additional granular details about the procedure performed.

In total, a table of 14,444 rows and 316 columns was obtained. A full list of features in the ACS-NSQIP database and the targeted vascular module can be found online in the NSQIP Participant Use Data Files (PUFs) [21]. Features were reduced to 80 demographic, clinical, and laboratory features according to clinical advice, 56 of which were feature variables, and the rest were outcomes. Feature variable used are listed in **S2 Table.** The data was split into training (9,429 cases from 2011–2015), validation (1978 cases from 2016), and independent testing (5,037 cases from 2017–2018). The training, validation, and testing set years were selected to allow a large training set size while leaving sufficient cases of death and readmission for evaluation.

76 out of 80 features contained missing values. Missing values in the ACS-NSQIP data have been found to be missing not at random [20], and therefore removal of patients with incomplete data may introduce unwanted bias. For this reason, missing values were imputed using Optimal Imputation, an imputation method that has demonstrated statistically significant gains in performance over state-of-the-art optimization methods [21].

Extraneous features may reduce model performance by leading to overfitting [22]. To avoid this problem, we selected features for inclusion in the machine learning model using Minimum Redundancy Maximum Relevance (mRMR), a method that selects features that are maximally relevant to the outcome and minimally redundant with other selected features [23]. To select the optimal number of features, we applied an incremental feature selection algorithm: for each k, where k is a value from 1 to the total number of predictive features (non-outcomes), mRMR was used to select features and develop a new model, and the new model was tested on the validation set. The model that returned the highest area under the receiver operating characteristic curve (AU-ROC), a measurement of a model’s ability to discriminate between classes, was selected. This algorithm was applied for both death and readmission outcomes separately, and it was applied for each of the four machine learning models tested.

Class imbalance can decrease model performance and lead to over-classification of the majority class [24]. To resolve this problem, for both death and readmission models, oversampling of the minority class was performed with ADASYN [25], a method that generates synthetic examples of the minority class.

To determine optimal hyperparameters for each model, we employed a grid search optimized for area under the receiver operating characteristic curve (AU-ROC). Parameters tuned include: For SVM—regularization parameter and gamma, for Random Forest—number of estimators, tree depth, maximum number of features, minimum number of samples required to split a node, and minimum number of samples required at each leaf node, for XGBoost—learning rate, maximum tree depth, minimum sum of instance weight needed in a child, gamma, subsample ratio of the training instances, and L1 regularization term, and for MLP—the size and number of hidden layers, the activation function, the L2 regularization term, and the learning rate.

The model was evaluated using the metrics area under the receiver operating characteristic curve (AU-ROC), sensitivity, and specificity. The model was interpreted using two methods: Gini impurity and SHapley Additive exPlanations (SHAP). Gini impurity measures the average reduction in impurity at splits in the decision tree—in other words, the ability of each feature in the random forest to split a group of mixed labeled cases into two pure class groups. SHAP is a game-theoretic approach to explain model predictions by generating models with different coalitions of features to determine the contribution of each feature to the final prediction. This allows for both global and local interpretation of the models [26].

To ensure that our model is equally accurate for different demographic groups, the test set was stratified by race (white and non-white), sex (male and female), and age (≥65 years and <65 years). The random forest models we developed for 30-day mortality and 30-day readmission were used to predict risk of death and readmission for each subgroup. AUC, accuracy, sensitivity, and specificity were used to evaluate the models. To compare the performance of the model within each demographic category and identify statistically significant differences in performance, we applied DeLong’s test for AU-ROC curve difference [27].

We extracted survival times from all cases including training, validation, and testing. We stratified all cases based on the top identified predictors according to Gini impurity. For 30-day mortality this included physiologic high-risk factors, elective surgery, functional status, HCT, creatinine, and INR. For 30-day readmission this included open wound/wound infection, major reintervention of treated arterial segment, elective surgery, claudication, and diabetes. We then performed Kaplan-Meier analysis on subgroups for each predictor. The log-rank test was used to test for significant differences in survival among patient subgroups for each predictor.

**Fig 1:**
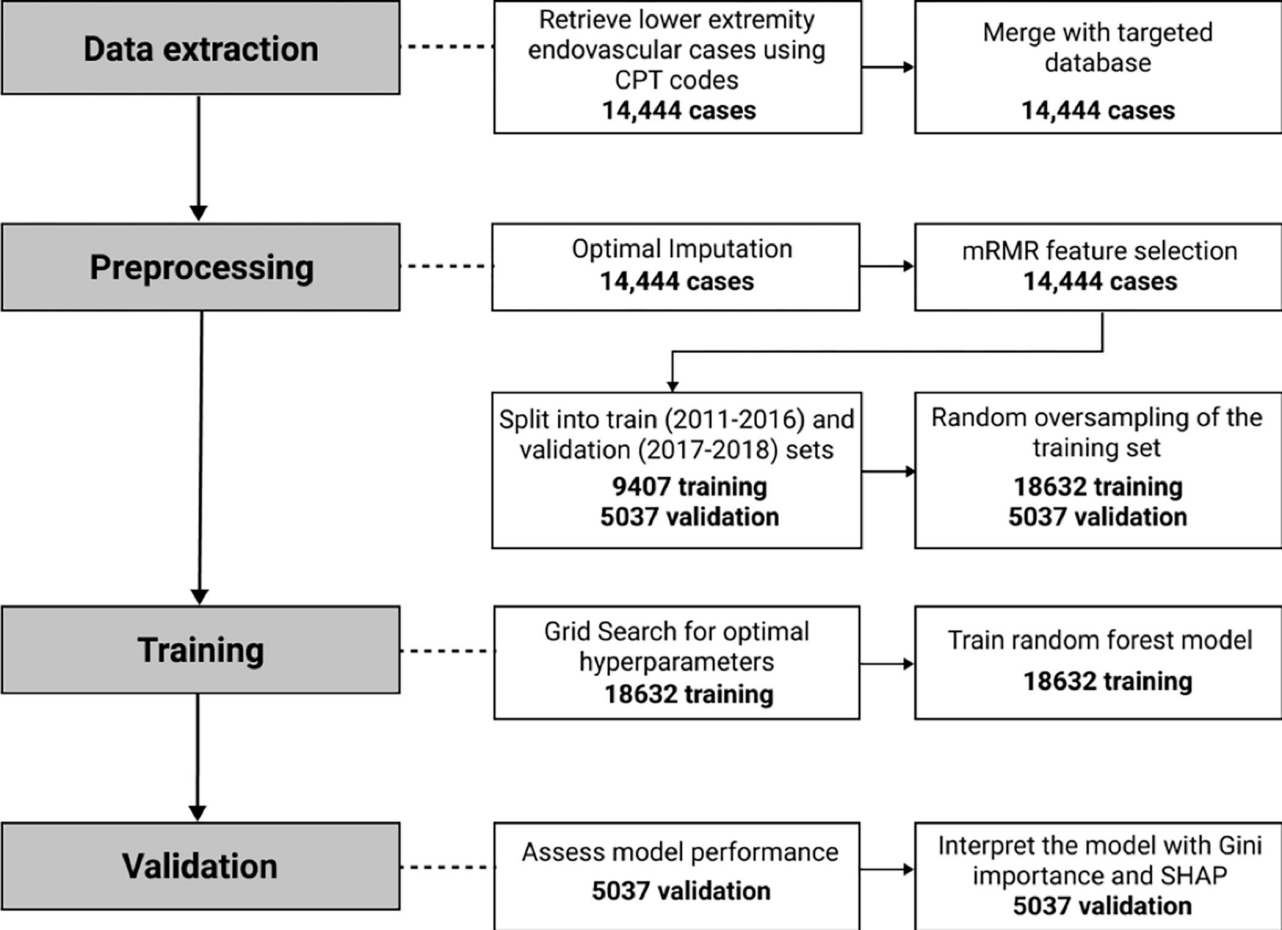
Flow chart illustrating the phases of model development.

The model was developed and evaluated using Python version 3.7.2 (packages: scikit-learn, interpretableai, pymrmr, imblearn, shap, lifelines, pandas, numpy, matplotlib) and R (packages: pROC [28]). The full procedure is illustrated in **Fig 1.** This study was approved by the Mass General Brigham Institutional Review Board. The data were analyzed anonymously and consent was waived for this study.

## Results

### Cohort

14,444 patients were included in this study. **Table 1** provides characteristics of the training and testing cohort. Overall, the mean (SD) age of patients included in model development was 69.1 (11.4) years. The majority of patients in the cohort were male (59%), and over 80% of patients were classified as ASA class 3 or higher. 83.1% had a cardiac comorbidity (use of hypertensive medication or history of chronic heart failure ≤30 days prior to surgery) and 10% had a renal comorbidity (acute renal failure ≤24 hours, or use of renal replacement therapy ≤2 weeks prior to procedure). 53.3% of the cohort had diabetes. 138 (1%) patients died and 1,699 (11.8%) were readmitted to the hospital within 30 days.

**Table 1:** Patient population demographics.

				Testing	Training				p
n				5037	9407				
			Clinical Comorbidities and Presenting Symptomatology						
Obesity (%)				1737 (34.4)	2956 (31.4)				<0.001
Diabetes (%)				2847 (56.5)	6123 (54.3)				0.010
Recent Smoking History (%)				1426 (29.0)	2922 (30.0)				0.222
Pulmonary Comorbidity (%)				522 (10.4)	1010 (10.7)				0.487
Cardiac Comorbidity (%)				4234 (84.1)	8033 (85.4)				0.032
Dyspnea (%)				544 (10.8)	1020 (10.8)				0.937
Recent Steroid Use (%)				272 (5.4)	593 (6.3)				0.029
Renal Comorbidity (%)				501 (9.9)	950 (10.1)				0.771
Emergent Procedure (%)				194 (3.9)	367 (3.9)				0.782
ASA Class 3–5 (%)				4701 (93.3)	8231 (87.4)				<0.001
Age (mean (SD))				69.4(11.2)	68.9 (11.5)				0.012
Asymptomatic (%)				272 (5.4)	492 (5.2)				0.664
Claudication (%)				1523 (30.2)	2960 (31.5)				0.128
Critical Limb Ischemia w/ Rest Pain (%)				933 (18.5)	1863 (19.8)				0.063
Critical Limb Ischemia w/ Tissue Loss (%)				2172 (43.1)	3767 (40.0)				<0.001
Symptoms Unknown (%)				86 (1.7)	126 (1.3)				0.080
Dependent Functional Status (%)				612 (12.1)	965 (10.2)				<0.001
Bleeding Disorder (%)				1624 (32.2)	3082 (32.8)				0.523
Recent Weight Loss (%)				44 (0.9)	82 (0.9)				0.991
**Demographics**				**Testing**	**Training**				
Male (%)				3080 (61.1)	5443 (57.9)				0.002
Female (%)				1906 (37.8)	3765 (40.0)				0.002
American Indian or Alaska Native (%)				17 (0.3)	33 (0.4)				0.896
Asian (%)				79 (1.6)	138 (1.5)				0.633
Black or African American (%)				1003 (19.9)	1701 (18.1)				0.007
Native Hawaiian or Pacific Islander (%)				6 (0.1)	8 (0.08)				0.531
Race Unknown (%)				653 (13.0)	999 (10.6)				<0.001
White (%)				3279 (65)	6519 (69.3)				<0.001
**Pre-Op Labs**				**Testing**	**Training**				
Sodium (mean (SD))				138 (3.2)	138.2 (3.3)				<0.001
BUN (mean (SD))				23.7 (14.0)	23.2 (13.5)				0.035
INR (mean (SD))				1.15 (0.34)	1.15 (0.32)				0.406
PTT (mean (SD))				35.8 (12.2)	35.6 (12.5)				0.546
Creatinine (mean (SD))				1.63 (1.7)	1.63 (1.7)				0.982
Albumin (mean (SD))				3.59 (0.53)	3.62 (0.52)				0.002
Bilirubin (mean (SD))				0.55 (0.27)	0.56 (0.35)				0.040
WBC (mean (SD))				8.36 (1.0)	8.27 (3.8)				0.167
HCT (mean (SD))				36.4 (6.2)	36.5 (6.0)				0.116
ALKPHOS (mean (SD))				96.9 (42.2)	96.0 (52.8)				0.294
Platelet count (mean (SD))				249 (92.4)	241 (87.8)				<0.001
Death (%)				47 (0.01)	91 (0.01)				0.840

### Mortality

**Fig 2:**
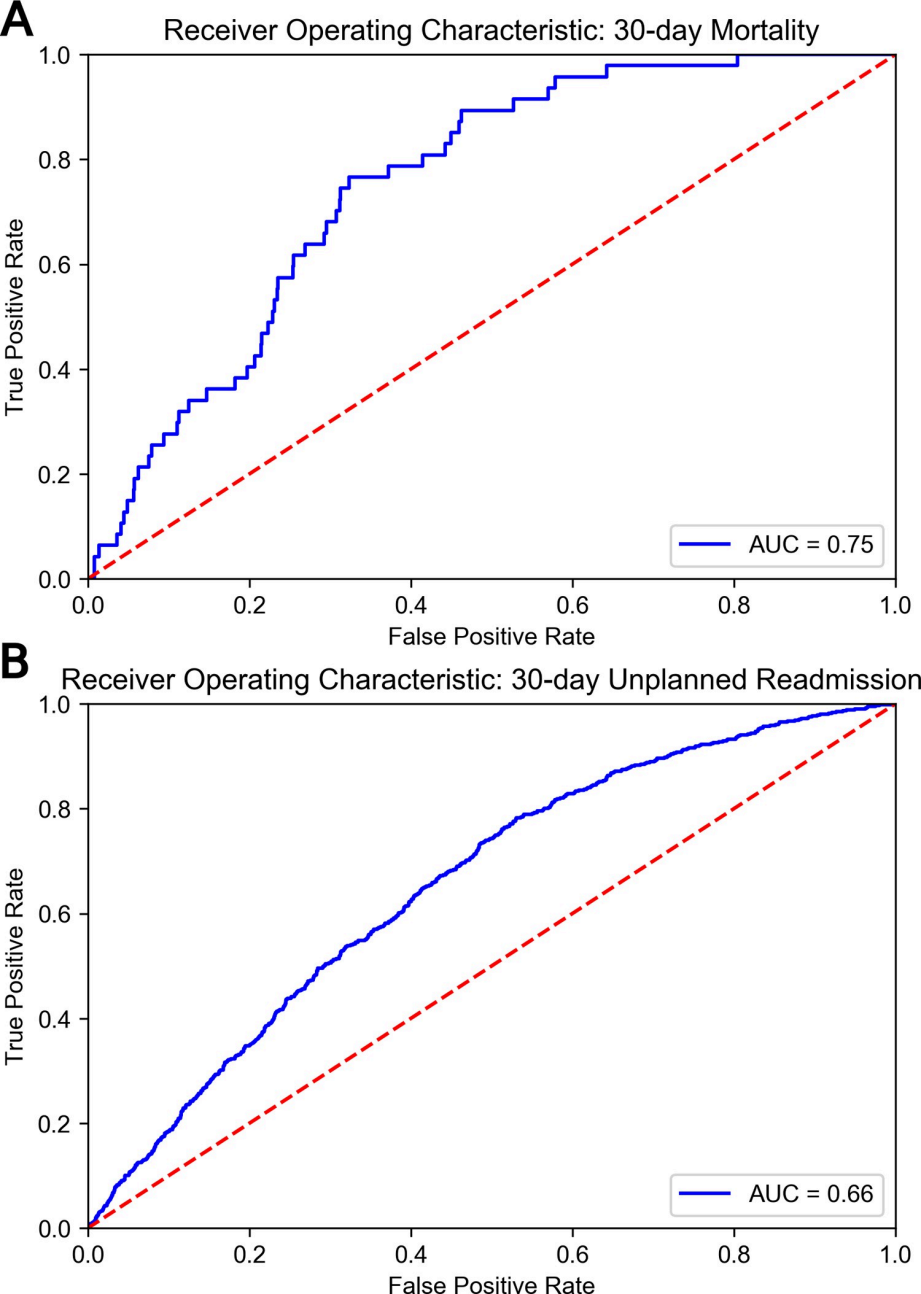
Receiver-operating characteristic curves for a) 30-day mortality and b) 30-day unplanned readmission models.

For 30-day procedure-related mortality prediction, our best-performing machine learning model was the random forest, which achieved an AU-ROC of 0.75 (95% CI: 0.71–0.79), accuracy of 0.87 sensitivity of 0.77 and specificity of 0.68 on the test set. On the training set, the model achieved an AU-ROC of 0.89, accuracy of 0.88, sensitivity of 0.69, and specificity of 0.88. The results of all models on the testing set are shown in **Table 2.** The AU-ROC curve is pictured in **Fig 2A**. 18 features are used in this model: Physiologic high-risk factors, hematocrit, blood urea nitrogen (BUN), creatinine, claudication, albumin, age, elective surgery designation, white blood cell count, serum glutamic oxaloacetic transaminase (SGOT), international normalized ratio (INR), alkaline phosphatase, bilirubin, platelet count, functional status, renal comorbidities, dyspnea, and open wound or wound infection.

**Table 2:** Performance metrics of all models on the testing set.

	AUC	Accuracy	Sensitivity	Specificity
Random Forest	0.75	0.87	0.77	0.68
Support Vector Machine (SVM)	0.75	0.70	0.84	0.49
Multilayer Perceptron (MLP)	0.72	0.97	0.09	0.98
Extreme Gradient Boosting (XGBoost)	0.74	0.92	0.76	0.49

The random forest model is an ensemble machine learning model consisting of many individual decision trees that are generated independently of one another, and the output of all decision trees are averaged to make a prediction. The trees are generated by taking subsamples of the dataset and finding optimal features to split on to minimize node impurity. The impurity criterion used for the random forest is Gini Impurity, calculated as the following:

$$Gini\;impurity=1-[{\left(P_{1}\right)}^{2}+{\left(P_{0}\right)}^{2}]$$

Where P_1_ is the probability of a death or readmission class and P_0_ is the probability of a non-death or readmission class. A weighted Gini impurity is calculated with the following equation:

$$Weighted\;Gini\;impurity=w_{i}G_{i}-w_{l}G_{l}-w_{r}G_{r}$$

Where w_i_ is the weight of the current node, G_i_ is the Gini impurity of the current node, w_l_ is the weight of the left node, G_l_ is the Gini impurity of the left node, w_r_ is the weight of the right node, and G_r_ is the Gini impurity of the right node.

**Fig 3:**
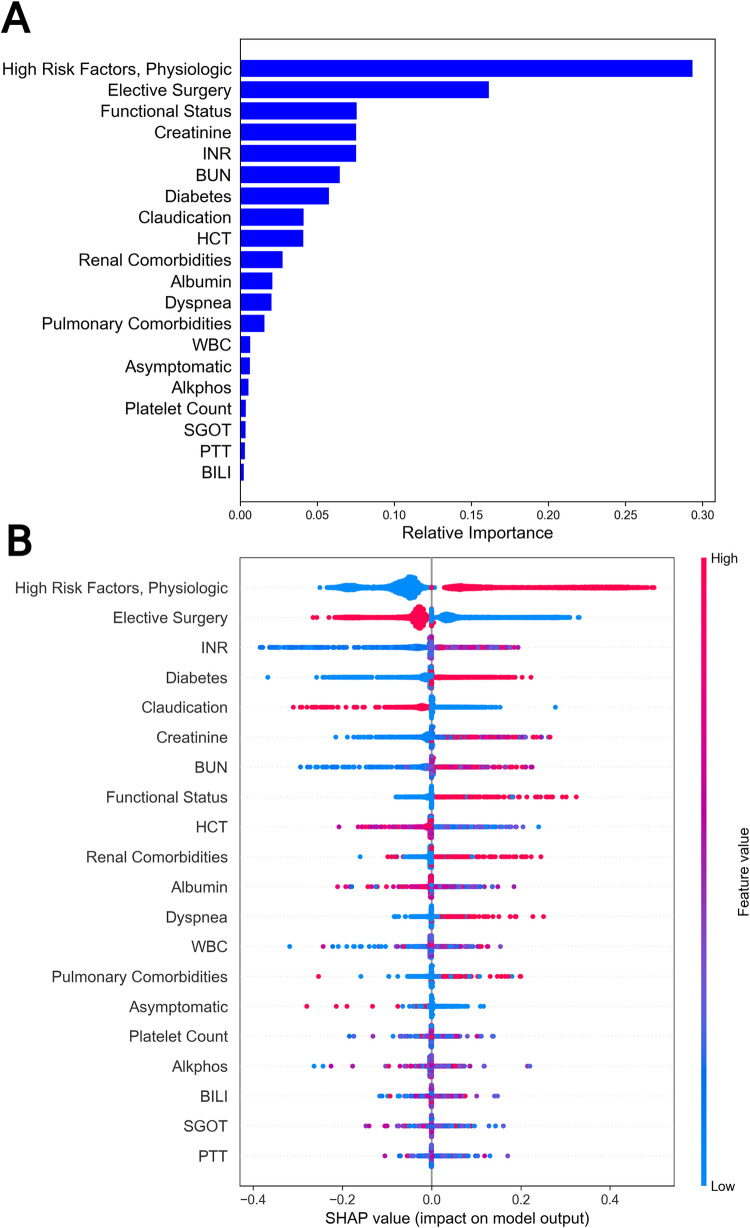
Gini impurity and SHAP scores for mortality. a) Gini impurity scores for features included in the 30-day mortality model. Higher values indicate increased effectiveness of features at separating those at risk of 30-day mortality from those not at risk of 30-day mortality. b) A Shapley summary plot. Color indicates feature value (red: High, blue: Low) and position.

To interpret the model, we used both Gini impurity and Shapley Additive exPlanations (SHAP). Gini impurity enables us to understand feature relevance at a global level, while a SHAP summary plot allows us to visualize feature contributions at the individual patient level and overall feature contribution patterns. According to Gini impurity values, the most important predictors of 30-day mortality were physiologic high-risk factors (New York Heart Association class III/IV congestive heart failure, left ventricular ejection fraction < 30%, unstable angina, or myocardial infarction within 30 days), elective surgery, functional status, hematocrit (HCT), and creatinine. A full list of features in order of importance as determined by Gini impurity is pictured in **Fig 3A**. The most important predictors identified by SHAP were physiologic high-risk factors, elective surgery, INR, diabetes, and claudication. The SHAP summary plot depicting feature importance and the direction of feature influence is shown in **Fig 3B.**

### Readmission

For 30-day unplanned readmission prediction, our best performing model was also the random forest model, which attained an AU-ROC of 0.69 (95% CI: 0.67–0.71), sensitivity of 0.76, and specificity of 0.55. The results of all models are shown in **Table 3.** The AU-ROC curve is pictured in **Fig 2B**. 16 features are used in this model: Major reintervention of treated arterial segment, hematocrit, albumin, claudication, creatinine, critical limb ischemia with tissue loss, open wound or wound infection, renal comorbidities, alkaline phosphatase, INR, BUN, physiologic high risk factors, elective surgery designation, diabetic status, American Society of Anesthesiologists (ASA) physical status greater than 3, and sodium.

**Table 3:** Performance metrics of all readmission models on the testing set.

	AUC	Accuracy	Sensitivity	Specificity
Random Forest	0.68	0.73	0.70	0.59
Support Vector Machine (SVM)	0.62	0.57	0.65	0.55
Multilayer Perceptron (MLP)	0.65	0.56	0.66	0.55
Extreme Gradient Boosting (XGBoost)	0.68	0.87	0.68	0.61

**Fig 4:**
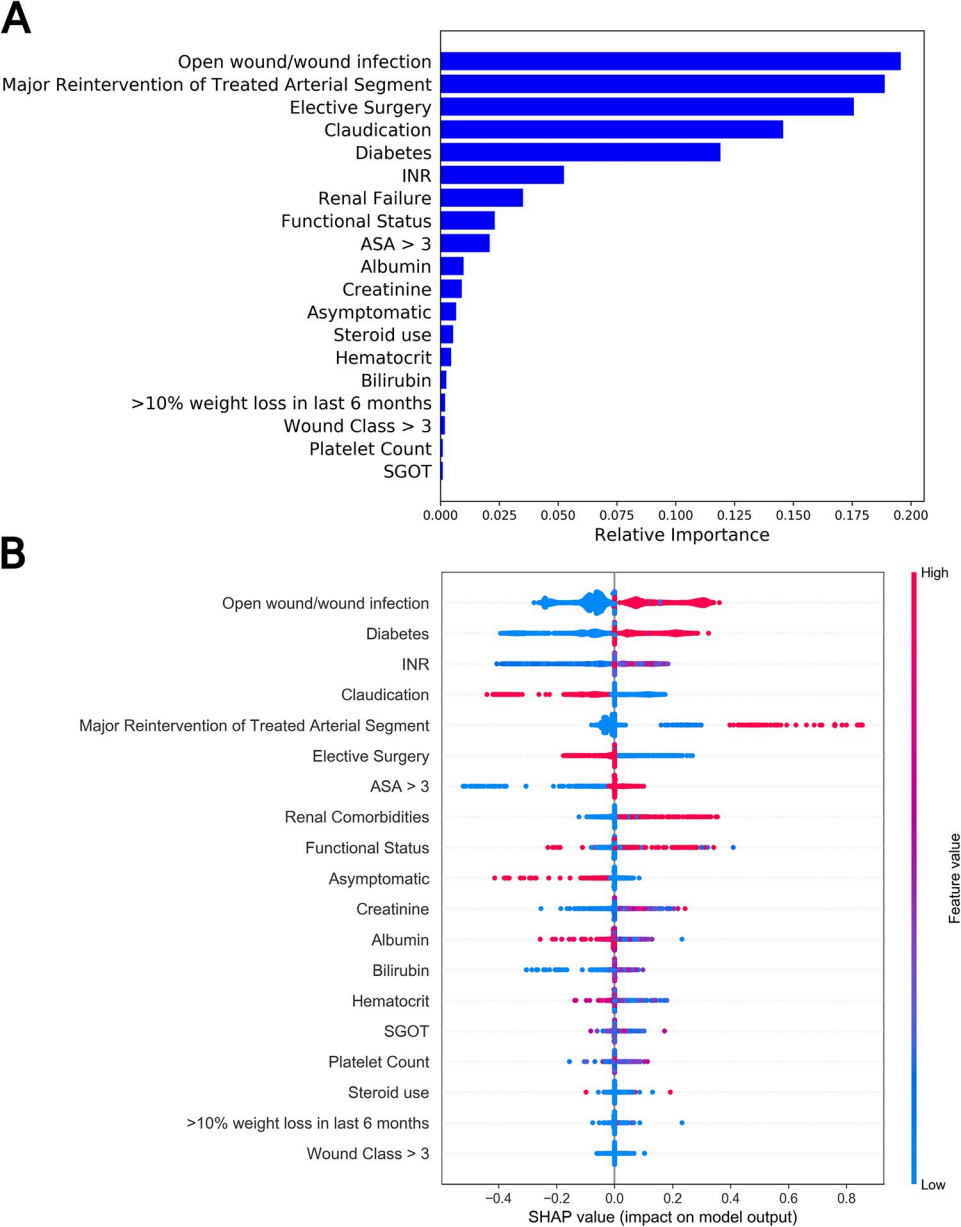
Gini impurity and SHAP scores for unplanned readmission. a) Gini impurity scores for features included in the 30-day unplanned readmission model. Higher values indicate increased effectiveness of features at separating those at risk of 30-day unplanned readmission from those not at risk of 30-day unplanned readmission. b) A Shapley summary plot. Color indicates feature value (red: High, blue: Low) and position along the x-axis indicates magnitude and direction of the feature’ impact on model predictions.

According to Gini impurity values, the most important predictors of unplanned readmission include open wound/wound infection, major reintervention of treated arterial segment, elective surgery, claudication, and diabetes. A full list of features in order of Gini impurity is pictured in **Fig 4A**. The most important features according to SHAP were open wound/wound infection, diabetes, INR, claudication, and major reintervention of treated arterial segment. The SHAP summary plot is shown in **Fig 4B.** Evaluation metrics for both mortality and readmission are shown in **Table 4**.

**Table 4:** AUC, accuracy, sensitivity, and specificity of the random forest models.

Mortality					Readmission			
	AUC	Accuracy	Sensitivity	Specificity	AUC	Accuracy	Sensitivity	Specificity
*Training Data*	0.89	0.88	0.69	0.88	0.71	0.76	0.42	0.80
*Testing Data*	0.75	0.87	0.77	0.68	0.68	0.73	0.70	0.59
*White*	0.77	0.87	0.62	0.75	0.69	0.74	0.62	0.64
*Non-white*	0.70	0.86	0.67	0.69	0.67	0.71	0.75	0.56
*Male*	0.75	0.85	0.63	0.73	0.70	0.72	0.69	0.60
*Female*	0.76	0.88	0.65	0.75	0.67	0.76	0.61	0.65
*Age > = 65*	0.75	0.85	0.66	0.69	0.67	0.76	0.60	0.63
*Age < 65*	0.72	0.89	0.58	0.79	0.71	0.71	0.79	0.58

### Fairness

**Fig 5:**
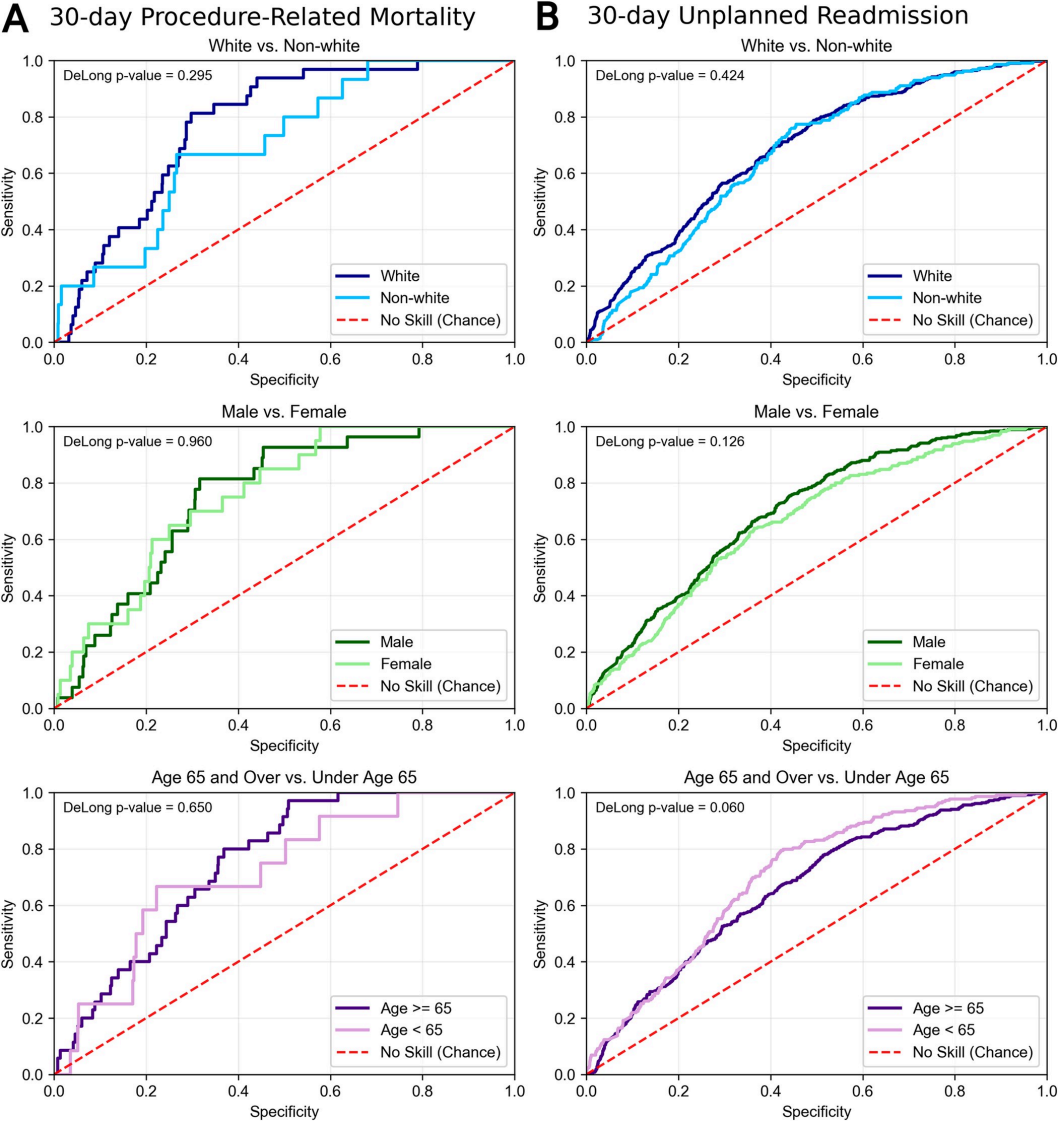
Model performance on demographic subgroups of the test set, demonstrating equivalent performance on race (white and non-white), sex (male and female), and age (under age 65 and 65 and older) groups.

DeLong’s test comparing the two models’ performance on racial, gender, and age subgroups indicates that both models perform equally well on white and non-white patients (30-day mortality: DeLong p-value = 0.322, 30-day readmission: DeLong p-value = 0.939), male and female patients (30-day mortality: DeLong p-value = 0.804, 30-day readmission: DeLong p-value = 0.130), and age ≥ 65 and < 65 years (30-day mortality: DeLong p-value = 0.804, 30-day readmission: DeLong p-value = 0.130). AU-ROC curve comparisons for each subgroup are pictured in **Fig 5.**

### Survival

Survival analysis on subgroups obtained from the top five identified predictors for 30-day mortality by Gini impurity shows significant differences in survival time between those who had physiological high-risk factors (p < 0.001), those who underwent elective versus non-elective procedures (p < 0.001), those who were independent versus those who were totally or partially dependent (p < 0.001), HCT <30% versus HCT of 30% or greater (p < 0.001), and those who had serum creatinine at levels indicating CKD stage 1 or 2 versus those with serum creatinine at levels indicating CKD stage 3 or above (p < 0.001). Survival analysis of subgroups obtained from the top five most important predictors for 30-day readmission shows significant differences in readmission time between those who had an open wound/wound infection versus those who did not (p < 0.001), those who were undergoing a major reintervention of the treated arterial segment versus those who were not undergoing reintervention (p < 0.001), those underwent elective surgery versus non-elective procedures (p < 0.001), those with claudication versus those without claudication (p < 0.001), and those with diabetes versus those without diabetes (p < 0.001). Survival curves for 30-day mortality and 30-day unplanned readmission are pictured in **S1 Fig.**

## Discussion

In this study, we have developed two machine learning models to stratify risk in PAD patients undergoing lower extremity endovascular procedures: one to identify patients at risk of 30-day procedure-related mortality and the other to identify patients who will be readmitted to the hospital within 30 days. The models were shown to perform well for both death (AUC: 0.75) and unplanned readmission (AUC: 0.68). We interpreted the models using Gini impurity and SHAP in order to gain an understanding of the importance and direction of influence of each feature. Among patients undergoing lower extremity endovascular procedures, the most important predictor of death was physiologic high-risk factors. The most important predictor of readmission was an open wound or wound infection. For each of the top five features for each outcome, we split the cohort into subgroups and performed survival analysis, including the log-rank test to identify differences between curves. We also demonstrated that the model is fair toward different demographic groups by stratifying the test set by race, sex, and age and evaluating the model on each group, yielding equivalent performance within each demographic category.

We compared the performance of several different machine learning models on the task of predicting 30-day procedure-related mortality and 30-day unplanned readmission: SVMs, XGBoost, Random Forests, and MLPs. For mortality, the random forest and SVM performed similarly well on the AU-ROC metric, but the specificity of the SVM and XGBoost models were both low at 0.49. A low specificity value indicates that the SVM model generated a large number of false positives, which can potentially mislead patients and clinicians when selecting interventions. Conversely, the multilayer perceptron achieved high specificity but low sensitivity, demonstrating an inability to perform the key task of identifying patients at risk of death. For readmission, the random forest model achieved the highest AUC at 0.68, with a balance of good sensitivity and specificity compared to the SVM and MLP, which both have low specificity at 0.55. The random forest and XGBoost models perform similarly, with only slight differences in performance. These results indicate that tree-based methods may be most suitable for the purpose of predicting mortality and readmission, possibly due to the ability of tree-based methods to consider nonlinear associations between variables in the ACS-NSQIP database.

Several models currently exist for predicting death and unplanned readmission for patients undergoing lower extremity infrainguinal endovascular interventions [5,11,12] and machine learning models have been developed for death and readmission prediction following other types of procedures and medical events [29–33]. Models for predicting mortality and unplanned readmission for patients undergoing infrainguinal endovascular interventions specifically, however, have been largely limited to multivariate logistic regression models, which may fail to account for nonlinear relationships in the data. These studies also focus solely on identifying risk factors for mortality and unplanned readmission, whereas our model, which outputs a numerical risk score, is able to be used for clinical decision-making in addition to understanding predictive factors for mortality and unplanned readmission risk. Using the numerical risk score for mortality and readmission as generated by our models, clinicians may be able to optimize treatment decisions as well as identify which patients are at the highest risk of readmission to implement strategies to reduce this risk.

Model interpretability is important for machine learning applications for medical decision-making, as model transparency is one of the major practical issues surrounding the implementation of AI into clinical workflows [34]. Model interpretability methods also enable clinicians to uncover novel clinical insights from machine learning models, such as predictors for death and readmission that are currently underappreciated in the clinic. Our model uses mRMR, which allows us to identify the features most highly correlated with death and readmission before model development, and we use both local (SHAP) and global (SHAP, Gini) explanatory methods to understand both the importance of each feature across all predictions as well as the direction of influence of each feature for individual predictions post-development. The most important variables we have identified align with current knowledge about the causes of death and readmission, which further highlights the usefulness of our model. For example, physiologic high-risk factors are the most important predictor for mortality. These high-risk factors include New York Heart Association class III/IV congestive heart failure, left ventricular ejection fraction < 30%, unstable angina, or myocardial infarction within 30 days. The association between PAD and cardiovascular mortality is well-known [35,36], corroborating our model’s identification of physiologic high risk factors as the most important predictor. Another important predictor for mortality, elective surgery designation, also demonstrates the clinical relevance of the model, as patients undergoing elective procedures are likely to have a more thorough pre-operative evaluation and optimization while having inherently less severe disease. Functional status, another predictor for mortality, has been shown to be associated with mortality in other studies of surgical outcomes [37–40] For readmission prediction, the identification of an open wound or wound infection as the most important feature also aligns with current knowledge, as an open wound and/or wound infection has been associated with readmission in multiple previous studies of surgical outcomes [11,41,42]. Another important predictor for readmission was a major reintervention of the treated arterial segment, which is a known predictor of readmission, as complex procedures which result in complications often require multiple admissions to manage the sequela of the initial complication. Our model emphasizes the importance of these variables and others while also allowing us to identify factors that have been previously underutilized such as alkaline phosphatase and INR. Our model recognizes correlations between these variables and 30-day mortality and readmission outcomes that—though not necessarily causal—may be useful as indicators of death and readmission risk. The survival analysis further highlights the importance of these features by demonstrating a statistically significant difference between subgroups of patients stratified by feature values.

The integration of machine learning models into healthcare settings has the potential to perpetuate pre-existing biases in the data and widen health disparities [43–45]. Therefore, it is important to ensure fairness to different demographic groups during the development of machine learning models for healthcare. Fairness is a complex social and mathematical concept with multiple conflicting definitions [46], and the problem of ensuring fairness in machine learning may not be solvable by computation alone [47]. Therefore, our demonstration of fairness—in this case demographic parity—must be understood as one part of an investigation into a complex web of biological and social factors impacting mortality and unplanned readmission in this patient population. The equal treatment of our model for all demographic groups is promising, and due to the complexity of fairness, is also a critical area for further study.

Another area of further study includes identifying modifiable risk factors for the prevention of death and readmission, as well as identifying effective strategies to target these factors to reduce mortality and unplanned readmission in patients undergoing lower extremity endovascular interventions for PAD. It may also be useful to further analyze underutilized variables that have been selected as predictors of mortality and readmission to determine their utility as markers of death and readmission risk. Future research also includes external validation of this model. As the next step, we propose a shadow evaluation method to test the model against real data without interfering with clinical decisions, a method that has been proposed in previous literature discussing translation of machine learning models in healthcare [48,49]. With this method, the model will output individual risk scores for death and readmission for each patient which will not be revealed to clinicians but later compared to 30-day outcomes to evaluate the model’s effectiveness. The model should also be tested on other datasets containing patients undergoing lower extremity endovascular interventions. This work may additionally be enhanced by the inclusion of time series data, which could facilitate the use of Long Short-Term Memory Networks (LSTM) to incorporate changes over time and improve performance.

There are several limitations to this study. The ACS-NSQIP database is implemented mostly at large teaching hospitals that have more quality-related accreditations and financial resources to conduct data collection [50]. Therefore, the data in the ACS-NSQIP database may not be representative of all surgical cases in the United States. Additionally, the ACS-NSQIP database tracks only 30-day outcomes, which prevents analysis on longer-term mortality and unplanned readmission. ACS-NSQIP also does not include all potentially relevant clinical variables such as vessel intervened, operator experience, and differences in procedure performance. Another limitation lies in the CPT code filtering process, which assumes equivalence between all procedures performed. Furthermore, as evidenced by the differences between training and testing model performance, our model somewhat overfits the training data. This was possibly caused by the data imputation step generating synthetic examples from a limited number of known observations [21,51]. However, our models perform well on an independent test set, indicating that this issue may be of limited concern. The feature selection and interpretation steps of our model with SHAP establish only a correlation between the selected variables and the studied outcomes and cannot be used to establish causal relationships.

## Conclusion

In conclusion, we have developed random forest models to output risk scores for 30-day mortality and 30-day unplanned readmission in patients undergoing lower extremity endovascular infrainguinal interventions for peripheral arterial disease. These models may help us personalize the medical decisions of patients with PAD to reduce the risk of mortality and readmission.

## Supporting information

S1 Fig Survival curves for subgroups stratified by top predictive features. (30-day mortality: Physiologic high-risk factors, elective surgery, functional status, HCT, and creatinine, 30-day unplanned readmission: Open wound/wound infection, major reintervention of treated arterial segment, elective surgery, claudication, and diabetes).
(TIF)

S1 Table CPT codes of cases extracted from the ACS-NSQIP database. (PDF)

S2 Table Variables used for model development. (PDF)
