# Supplementary figures and images for: Risk stratification with explainable machine learning for 30-day procedure-related mortality and 30-day unplanned readmission in patients with peripheral arterial disease
Source: PLoS One. 2022 Nov 21;17(11):e0277507. doi: 10.1371/journal.pone.0277507 (PMC9678279; doi:10.1371/journal.pone.0277507)

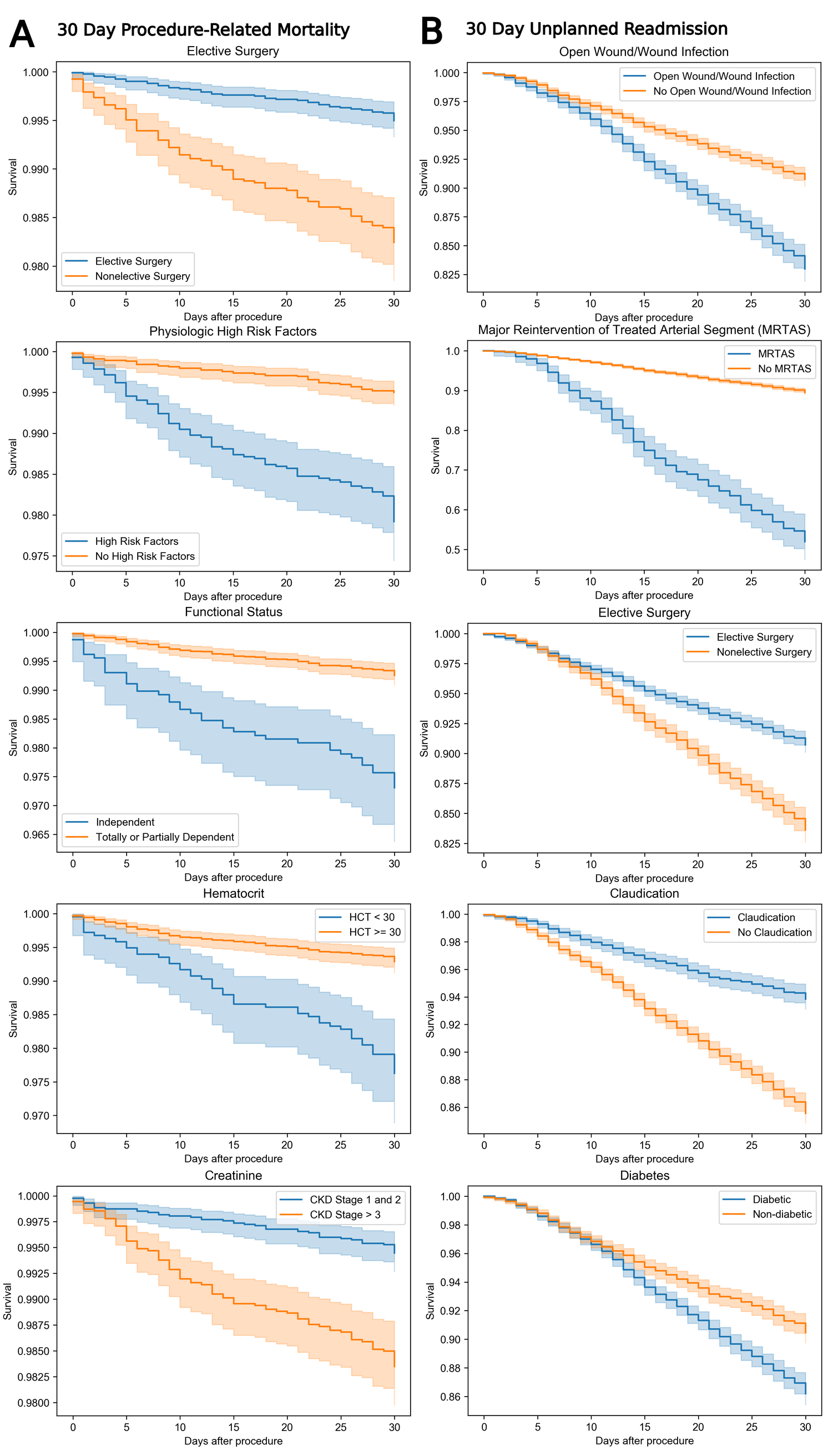

Supplement: S1 Fig — (30-day mortality: Physiologic high-risk factors, elective surgery, functional status, HCT, and creatinine, 30-day unplanned readmission: Open wound/wound infection, major reintervention of treated arterial segment, elective surgery, claudication, and diabetes). (TIF) [file pone.0277507.s001.tif]
