# Supplementary material for: Risk stratification with explainable machine learning for 30-day procedure-related mortality and 30-day unplanned readmission in patients with peripheral arterial disease
Source: PLoS One. 2022 Nov 21;17(11):e0277507. doi: 10.1371/journal.pone.0277507 (PMC9678279; doi:10.1371/journal.pone.0277507)
Supplement: S1 Table — (PDF) [file pone.0277507.s002.pdf]

CPT Code	Description
37224	Revascularization, endovascular, open or percutaneous, femoral, popliteal artery(s), unilateral; with transluminal angioplasty
37225	Revascularization, endovascular, open or percutaneous, femoral, popliteal artery(s), unilateral; with atherectomy, includes angioplasty within the same vessel, when performed
37226	Revascularization, endovascular, open or percutaneous, femoral, popliteal artery(s), unilateral; with transluminal stent placement(s), includes angioplasty within the same vessel, when performed
37227	Revascularization, endovascular, open or percutaneous, femoral, popliteal artery(s), unilateral; with transluminal stent placement(s) and atherectomy, includes angioplasty within the same vessel, when performed
37228	Revascularization, endovascular, open or percutaneous, tibial, peroneal artery, unilateral, initial vessel; with transluminal angioplasty
37229	Revascularization, endovascular, open or percutaneous, tibial, peroneal artery, unilateral, initial vessel; with atherectomy, includes angioplasty within the same vessel, when performed
37230	Revascularization, endovascular, open or percutaneous, tibial, peroneal artery, unilateral, initial vessel; with transluminal stent placement(s), includes angioplasty within the same vessel, when performed
37231	Revascularization, endovascular, open or percutaneous, tibial, peroneal artery, unilateral, initial vessel; with transluminal stent placement(s) and atherectomy, includes angioplasty within the same vessel, when performed
37232	Revascularization, endovascular, open or percutaneous, tibial/peroneal artery, unilateral, each additional vessel; with transluminal angioplasty
37233	Revascularization, endovascular, open or percutaneous, tibial/peroneal artery, unilateral, each additional vessel; with atherectomy, includes angioplasty within the same vessel, when performed (List separately in addition to code for primary procedure)
37234	Revascularization, endovascular, open or percutaneous, tibial/peroneal artery, unilateral, each additional vessel; with transluminal stent

	placement(s), includes angioplasty within the same vessel, when performed (List separately in addition to code for primary procedure)
