# Supplementary material for: Risk stratification with explainable machine learning for 30-day procedure-related mortality and 30-day unplanned readmission in patients with peripheral arterial disease
Source: PLoS One. 2022 Nov 21;17(11):e0277507. doi: 10.1371/journal.pone.0277507 (PMC9678279; doi:10.1371/journal.pone.0277507)
Supplement: S2 Table — (PDF) [file pone.0277507.s003.pdf]

**Features**
High Risk Factors, Physiologic
Pre-procedural Antiplatelet Medication
Pre-procedural Medication-Statin
Pre-procedural Medication-Beta Blocker
Wound Infection/Complication
Major Amputation (Transtibial or Proximal)
Sex
Age
Disseminated cancer
Systemic Sepsis
Pre-operative serum sodium
Pre-operative BUN
Pre-operative serum creatinine
Pre-operative serum albumin
Pre-operative total bilirubin
Pre-operative SGOT
Pre-operative alkaline phosphatase
Pre-operative WBC
Pre-operative hematocrit
Pre-operative platelet count
Pre-operative PTT
Pre-operative International Normalized Ratio (INR) of PT values
Pre-operative PT
Emergency case
Elective Surgery
Critical limb ischemia: tissue loss
Claudication
Critical limb ischemia: rest pain
Asymptomatic
Not documented
Major Reintervention of Treated Arterial Segment
American Indian or Alaska Native
Black or African American
Native Hawaiian or Pacific Islander
Asian
Unknown/Not Reported
White
BMI
Obese
Diabetes mellitus with oral agents or insulin
Current smoker within one year

Dyspnea
Functional health status Prior to Surgery
Ventilator dependent or History of severe COPD
Ascites or Heart failure (CHF) in 30 days before surgery or Hypertension requiring medication
Acute renal failure (pre-op) or Currently on dialysis (pre-op)
Open wound/wound infection
Immunosuppressive Therapy
Malnourishment
Bleeding disorders
Preop Transfusion of >= 1 unit of whole/packed RBCs in 72 hours prior to surgery
Contaminated or dirty/infected wound
ASA Classification > 3
